# Effects of Topical Icing on Inflammation, Angiogenesis, Revascularization, and Myofiber Regeneration in Skeletal Muscle Following Contusion Injury

**DOI:** 10.3389/fphys.2017.00093

**Published:** 2017-03-07

**Authors:** Daniel P. Singh, Zohreh Barani Lonbani, Maria A. Woodruff, Tony J. Parker, Roland Steck, Jonathan M. Peake

**Affiliations:** ^1^Tissue Repair and Regeneration Group, Institute of Health and Biomedical Innovation, Queensland University of TechnologyBrisbane, QLD, Australia; ^2^Biofabrication and Tissue Morphology Group, Institute of Health and Biomedical Innovation, Queensland University of TechnologyBrisbane, QLD, Australia; ^3^School of Biomedical Sciences, Queensland University of TechnologyBrisbane, QLD, Australia; ^4^Medical Engineering Research Facility, Queensland University of TechnologyBrisbane, QLD, Australia

**Keywords:** muscle injury, cryotherapy, regeneration, inflammation, angiogenesis

## Abstract

Contusion injuries in skeletal muscle commonly occur in contact sport and vehicular and industrial workplace accidents. Icing has traditionally been used to treat such injuries under the premise that it alleviates pain, reduces tissue metabolism, and modifies vascular responses to decrease swelling. Previous research has examined the effects of icing on inflammation and microcirculatory dynamics following muscle injury. However, whether icing influences angiogenesis, collateral vessel growth, or myofiber regeneration remains unknown. We compared the effects of icing vs. a sham treatment on the presence of neutrophils and macrophages; expression of CD34, von Willebrands factor (vWF), vascular endothelial growth factor (VEGF), and nestin; vessel volume; capillary density; and myofiber regeneration in skeletal after muscle contusion injury in rats. Muscle tissue was collected 1, 3, 7, and 28 d after injury. Compared with uninjured rats, muscles in rats that sustained the contusion injury exhibited major necrosis, inflammation, and increased expression of CD34, vWF, VEGF, and nestin. Compared with the sham treatment, icing attenuated and/or delayed neutrophil and macrophage infiltration; the expression of vWF, VEGF, and nestin; and the change in vessel volume within muscle in the first 7 d after injury (*P* < 0.05). By contrast, icing did not influence capillary density in muscle 28 d after injury (*P* = 0.59). The percentage of immature myofibers relative to the total number of fibers was greater in the icing group than in the sham group 28 d after injury (*P* = 0.026), but myofiber cross-sectional area did not differ between groups after 7 d (*P* = 0.35) and 28 d (*P* = 0.30). In conclusion, although icing disrupted inflammation and some aspects of angiogenesis/revascularization, these effects did not result in substantial differences in capillary density or muscle growth.

## Introduction

Closed soft tissue trauma or contusion injuries in skeletal muscle are relatively common among athletes (Canale et al., 1981; Volpi et al., 2004) and industrial workers (Dement and Lipscomb, 1999). These types of injuries typically occur when a sudden, heavy, compressive force is applied to the muscle and are therefore common in contact sports (Jarvinen et al., 2005). Contusion injuries are clinically manifested by localized pain, swelling, reduced range of motion, and tenderness to palpation (Kary, 2010). At the molecular level, contusion injuries result in transient necrosis, infiltration of inflammatory cells, and expression of proinflammatory cytokines. These processes are followed by the formation of new myofibers and subsequent myofiber maturation (Merrick et al., 1999; Lee et al., 2005; Schaser et al., 2006, 2007; Carvalho et al., 2010; Puntel et al., 2011; Takagi et al., 2011; Vieira Ramos et al., 2016).

The process of muscle healing begins soon after injury and involves phases of tissue destruction, repair, and remodeling. These phases involve regeneration of myofibers, formation of connective scar tissue, angiogenesis, and vascularization (Jarvinen et al., 2005; Ceafalan et al., 2015). Angiogenesis is a key process in muscle regeneration after injury (Ochoa et al., 2007; Miyazaki et al., 2011; Huey et al., 2016). Successful muscle regeneration depends on restoration of the vascular network required for the exchange of oxygen and nutrients, and the formation of mature muscle fibers (Rhoads et al., 2009). Following injury, angiogenesis is tightly coordinated with the proliferation, differentiation, and fusion of satellite cells to existing myofibers (Christov et al., 2007). Activated satellite cells accumulate and proliferate near capillaries and are stimulated to grow by various growth factors secreted by surrounding epithelial cells (Christov et al., 2007). Proliferating and differentiating satellite cells stimulate endothelial cells to join together to form nascent blood vessels (Christov et al., 2007) and microvascular fragments to form new sprouts (Rhoads et al., 2009). Angiogenesis in regenerating muscle tissue also depends on macrophages, chemokines such as monocyte chemotactic protein 1, and various growth factors and their receptors (Ochoa et al., 2007; Wagatsuma, 2007; Novak et al., 2011; Ceafalan et al., 2015).

A variety of options are used to treat muscle injuries including: immobilization/remobilization; rest, ice, compression, and elevation (RICE); ultrasound; hyperbaric oxygen therapy; anti-inflammatory or antifibrotic drugs; and treatment with platelet-rich plasma or muscle-derived stem cells. Icing is a mainstay treatment for many types of acute musculoskeletal injuries (Meeusen and Lievens, 1986). The general rationale for using ice to treat such injuries is to alleviate pain, reduce tissue metabolism, and modify vascular responses to restrict swelling (Swenson et al., 1996). A number of studies have investigated the effects of cryotherapy (e.g., icing or perfusion of cold saline) on muscle regeneration, inflammation, and oxidative stress after crush or contusion injuries (Merrick et al., 1999; Lee et al., 2005; Schaser et al., 2006, 2007; Carvalho et al., 2010; Puntel et al., 2011; Takagi et al., 2011; Vieira Ramos et al., 2016). Infusion of cold saline in injured skeletal muscle restores microvascular hemodynamics at 1 h (Schaser et al., 2006) and 24 h (Schaser et al., 2007) after injury.

To date, no research has investigated whether cryotherapy influences angiogenesis during the later phases of muscle growth/regeneration (i.e., >1 d after injury). It is important to understand how icing influences angiogenesis in skeletal muscle following injury for two reasons. First, icing is a common treatment for muscle injuries. Second, angiogenesis is closely linked with muscle repair. Evidence from other tissue types suggests that hypothermia can alter the secretion of vascular endothelial growth factor (VEGF) (Coassin et al., 2010; Takeyama et al., 2015) and angiogenesis (Kuo et al., 2010; Kao et al., 2011). It remains unknown whether hypothermia exerts similar effects in skeletal muscle tissue.

The aim of this study was to examine the effects of icing soon after muscle contusion injury on subsequent inflammation, angiogenesis, vessel volume, and myofiber regeneration. We hypothesized that icing would attenuate inflammation, angiogenesis, and changes in vessel volume, which may slow myofiber regeneration.

## Materials and methods

The experimental procedures in this project were approved by the Queensland University of Technology Animal Ethics Committee (ethics approval number 11-318). The experiments were conducted in accordance with the Australian Code of Practice for the Care and Use of Animals for Scientific Purposes (National Health and Medical Research Council, 2013).

### Experimental overview

**Figure 1:**
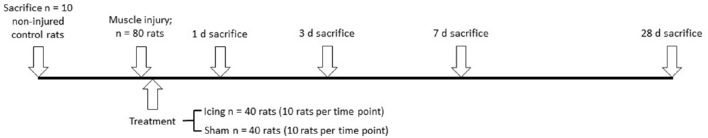
**Time course of experimental procedures**. The uninjured control, icing, and sham treatment groups at each time of sacrifice were divided into groups of six rats for histology and immunohistochemistry, and four rats for micro-CT imaging.

The time course of experimental procedures is outlined in Figure 1. Ninety adult male Wistar strain rats (12 weeks old) were acquired from Animal Resources Centre (Canning Vale, WA, Australia). Adult rats were chosen to minimize any growth effects on muscle tissue regeneration during the weeks following injury. Eighty rats were divided randomly into an icing group or a sham group (*n* = 40 per group). Both groups of animals were subjected to a contusion muscle injury, after which icing or a sham treatment was applied to the skin covering the injury site. The rats in each group were sacrificed at four separate time points: 1, 3, 7, and 28 d after injury (*n* = 10 per group at each time point). Ten rats were used as uninjured and untreated controls.

### Muscle contusion injury

Anesthesia was induced before injury by placing the rat in an induction chamber containing 5% isofluorane delivered in 2–3 l O_2_/min. Anesthesia was then maintained for at least 20 min after injury through a face mask that delivered 2–2.5% isofluorane in 1–1.5 L O_2_/min. This procedure was used so that the rats would not move while the ice or sham treatment was applied. Analgesia was achieved by subcutaneous injection of buprenorphine (0.05 mg/kg) immediately before injury and administration of tramadol (25 mg/l) in the drinking water during the first 5 d after injury. These anesthetic and analgesic treatments may have induced the expression of key mediators of angiogenesis, such as hypoxia-inducible factor-1α (HIF-1α) and VEGF (Li et al., 2006; Singleton et al., 2006; Luk et al., 2012). However, because we administered these treatments to all of the experimental animals, any such effects were most likely similar between the icing and sham groups.

During pilot work, we established a noninvasive contusion injury model using a custom-designed device based on an approach described by Stratton et al. (1984) that has been used extensively in other research. This pilot work confirmed that this approach caused substantial muscle contusion without perforating the skin or fracturing the bones of the hind limb. The procedure involved placing a rat with its left hind limb in an extended position on a platform. A cylindrical 400 g weight was placed in a metal column at a height 1.66 m above the platform and held in place temporarily with a metal pin. The weight was released by pulling the pin from the column. The weight then dropped onto the hind limb of the rat specifically in the region of the *biceps femoris* muscle. In this way, the injury was localized to a specific region of interest between the tibial notch of the knee joint and the tibiofibula conjunction.

### Icing and sham treatment

Icing treatment was administered based on the method described by Takagi et al. (2011). Five min after the contusion injury was induced and while the rat was still anesthetized, a 5-cm diameter cylindrical ice block was applied to the skin surrounding the injured muscle for 20 min. The ice block was massaged in a “**Figure 8**” motion on the injured area without compression. The underside of the leg was rested on an ice pack. In the sham group, a 50-ml flat-bottomed beaker (maintained at room temperature) was used to massage the injured area and to simulate the movement of the ice block, also for 20 min. These icing and sham treatments were applied once on the day of injury. Following icing or the sham treatment, the rats were free to move around their cages as they recovered from the muscle injury. At 1, 3, 7, or 28 d after injury, the rats were euthanized by CO_2_ asphyxiation.

### Histology and immunohistochemistry

Muscle biopsies from the *biceps femoris* muscle were fixed in 10% neutral buffered formalin for 1 d, processed, and then embedded in paraffin wax (Thermo Excelsior Tissue Processor and paraffin embedding station, Thermo Fisher Scientific, USA). Transverse serial sections (5 μm thick) were cut using a microtome (Leica Biosystems RM2235, Sydney, Australia) and mounted on poly-l-lysine adhesion glass slides (Thermo Fisher Scientific, Brisbane, Australia). Sections between a depth of 50 and 250 μm were used for analysis. Slides were stained with hematoxylin and eosin (H&E, Sigma-Aldrich, Sydney, Australia) for qualitative histological analysis of muscle regeneration. For immunohistochemistry, the slides were deparaffinized, treated with 3% H_2_O_2_ for 30 min, and then blocked with 2% bovine serum albumin (BSA) for 60 min. Heat-induced antigen retrieval was performed using trisodium citrate buffer (Sigma-Aldrich) for 40 min at 70°C in an oven (Cole-Parmer StableTemp oven, Sydney, Australia). The slides were incubated overnight at 4°C with the following primary antibodies: mouse monoclonal anti-rat granulocyte (HIS48, 1:50; BD Pharmingen, San Diego, CA, USA) to identify neutrophils; mouse monoclonal anti-CD68 (ED1) (1:500; Abcam, Cambridge, MA, USA) to identify macrophages; rabbit polyclonal anti-human VEGF A-20 (sc-152, 1:500; Santa Cruz Biotechnology, Santa Cruz, CA, USA) as a proangiogenic factor; rabbit monoclonal anti-CD34 (EP373Y, 1:500; Abcam) and rabbit polyclonal anti-human von Willebrand Factor (vWF) (ready-to-use; Dako, Carpinteria, CA, USA) to identify endothelial cells associated with capillaries (Qu et al., 1997; Niiyama et al., 2003; Fujino et al., 2005; Wiik et al., 2005; Ho et al., 2006; Hollemann et al., 2008); and mouse monoclonal anti-nestin (Rat-401, 1:400; Santa Cruz Biotechnology) as a marker of maturing endothelial cells in muscle (Cizkova et al., 2009b). The slides were then incubated for 40 min with a goat anti-mouse and anti-rabbit secondary antibody conjugated with horseradish peroxidase (Dako). Color was developed using 3,3′-diaminobenzidine (DAB Substrate Kit; Dako) followed by counter stain with Mayer's hematoxylin. The exclusion of the primary antibody as well an isotype control (mouse IgM isotype control; BD Pharmingen) served as negative controls.

### Image analysis

Slides were scanned using a Leica SCN400 slide scanner (Leica Microsystems, Wetzlar, Germany), and images were captured using Digital Image Hub software (Leica Microsystems). Briefly, at × 40 magnification, 10 fields of view were captured and quantified per slide for all six animals per treatment group and time point. ImageJ software was used to quantify areas of positive stain and to identify cells and tissue of interest. To quantify neutrophils and macrophages, positively stained cells were counted and are expressed as the number of cells per field of view. The 10 fields of view were averaged, and a single value was recorded per animal. The expression of CD34, vWF, VEGF, and nestin was assessed by the area of positive stain as a percentage of the total area of tissue within the field of view. Using this relative approach avoided problems caused by swelling of muscle fibers and endothelial cells, and allowed us to compare samples more reliably. Areas of muscle tissue with vessels with a diameter >100 μm and longitudinally oriented myofibers were excluded.

Cross-sectional area of muscle fibers was assessed by tracing the outline of 100 fibers using Digital Image Hub software to calculate the fiber area. Regenerating fibers were identified as those fibers with centrally located nuclei and are expressed as a proportion of the total number of fibers in 10 fields of view. Experimental and control groups were compared, and the numbers of inflammatory cells and extent of angiogenesis were graphically represented to determine effects of the ice treatment.

Capillary density was quantified by counting the number of CD34^+^ cells and fibers from five fields of view per slide at × 20 magnification using Digital Image Hub software. Capillary density is expressed as capillaries per fiber and capillaries per millimeters squared. As reported by Ochoa et al. (2007), necrosis in the tissue sections obtained at 1, 3, and 7 d after injury made it difficult to identify the borders of individual muscle fibers at these points. Accordingly, we could quantify capillary density only at 28 d after injury.

### Analysis of vessel volume

Quantitative, three-dimensional characterization of vessel volume in muscle tissue was performed using microcomputed tomography (micro-CT). This analysis was conducted on a separate group of 36 rats from those rats used for muscle histology and immunohistochemistry (see Figure 1).

Immediately after euthanasia, the vasculature was flushed with heparinized saline (0.9% normal saline and heparin sodium (100 U/ml), Pfizer Ltd, Sydney, Australia) using a peristaltic infusion pump (Pump 1: Cole-Parmer, 6-600 RPM, Extech Equipment Pty Ltd, Melbourne, Australia). Once the perfusate returned clear at the exit point at the right atrium, the blood vessels were then perfused with Microfil contrast agent (MV-122 Microfil kit, consisting of MV-compound, MV-diluent, and MV-curing agent; Flow Tech Inc., Carver, MA, USA), as described by Duvall et al. (2004), using a second peristaltic pump (Pump 2: 505 U, 220 RPM, Watson Marlow Ltd, NSW, Australia). The volume of contrast agent used per rat was 165 ml and comprised a compound–diluent ratio of 1:2 mixed with 10% curing agent (i.e., 50 ml compound, 100 ml diluent, and 15 ml curing agent). The contrast agent was pumped into the rat at a rate of 20 ml/min to keep the perfusion pressure low enough to prevent any vasculature breakage or leakage. Not all of the contrast agent remained within the vasculature because the right atrium was punctured to release the perfusion media after perfusion of the vasculature. However, the perfused volume and duration of perfusion ensured complete perfusion of the animal. This technique does not measure edema. Rather, because the contrast agent was limited to vessel space, no leakage of the contrast agent into extravascular space was observed by micro-CT or histological evaluation.

**Figure 2:**
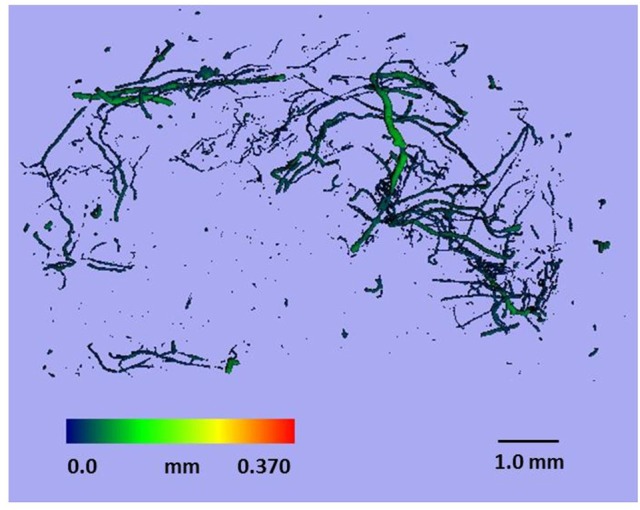
**Representative micro-CT image of the vascular network within a biopsy stack (250 slices) from one animal 1 d after injury**.

**Figure 3:**
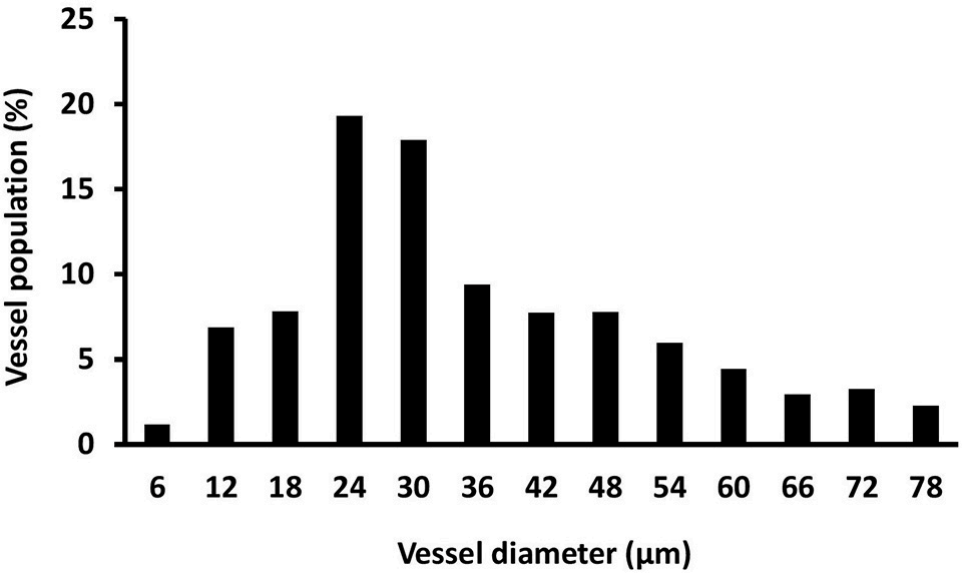
**Histogram showing the distribution of vessel size**. Vessel size was determined by micro-CT (vessel diameter 6–78 μm) within a biopsy stack (250 slices) from one animal 1 d after injury.

After perfusion, the rats were stored at 4°C overnight to allow the contrast agent to polymerize. The next day, the hind limbs were removed, and a tissue biopsy was taken from the region of interest (as described above) using an 8-mm disposable biopsy punch. This approach ensured that biopsies were obtained consistently from the same region of the muscle in each animal. The muscle biopsies were scanned using a micro-CT imaging system (μCT 40, Scanco Medical, Bassersdorf, Switzerland) and evaluated using Scanco software (μCT Evaluation Program, V6.5-3, Scanco Medical). The muscle biopsies were scanned at an energy of 55 kVp, intensity of 145 μA, and integration time of 250 ms, which resulted in a voxel size of 6 μm. The vascular network was segmented from the surrounding tissue using a lower threshold of 1,434 Hounsfield units and a low-pass Gaussian filter (σ = 0.8, support = 1). The total volume of the smaller blood vessels (<78 μm diameter) was quantified for the top 250 image slices of the biopsy scans. These data were used to calculate vessel volume. To reduce the potential variability within the micro-CT results, data from the injured rats was normalized relative to the uninjured rats (i.e., injured/uninjured × 100). Outliers in the vessel volume data were identified using the formula mean—(1.5 × SD) < measured value < mean + (1.5 × SD). Figure 2 shows a representative micro-CT image of the vascular network within a biopsy stack (250 slices) from one animal 1 d after injury. Figure 3 shows a histogram representing the distribution of vessels with diameter 6–78 μm within a biopsy stack (250 slices) from one animal 1 d after injury.

### Statistical analysis

All data were tested and confirmed as normally distributed using the Shapiro–Wilks formula. Two-factor analysis of variance was performed to determine if there were any main effects of time and group or group × time interactions. If any significant (*P* < 0.05) main effects were evident, unpaired *t*-tests were used to compare the control group vs. the icing and sham groups, and the icing vs. sham groups. This statistical analysis was performed using IBM SPSS Statistics (version 23, IBM, Armonk, NY, USA). The false discovery rate was used to control for multiple comparisons between groups. Cohen's effect size (*d*) was also calculated to assess treatment effects and was considered small (*d* < 0.2), moderate (*d* = 0.2–0.5), or large (*d* ≥ 0.8). All data are presented as mean ± SD.

## Results

**Figure 4:**
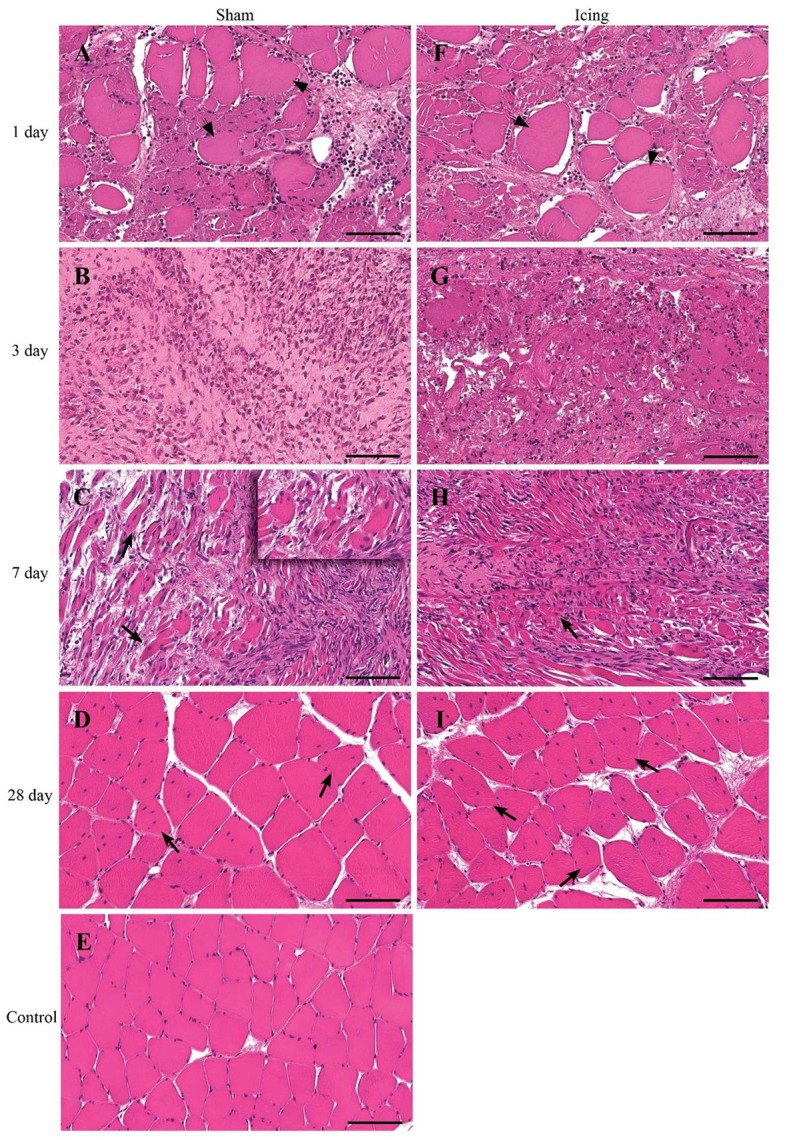
**Representative cross sections of skeletal muscle tissue**. Muscle tissue was stained with H&E in the sham group **(A–D)** and the icing group **(F–I)** at 1, 3, 7, and 28 d after injury. **(E)** Shows muscle tissue from an uninjured rat for comparison. Arrowheads indicate necrotic muscle fibers. Arrows indicate regenerating muscle fibers (centrally placed nuclei). Scale bar = 100 μm.

**Figure 5:**
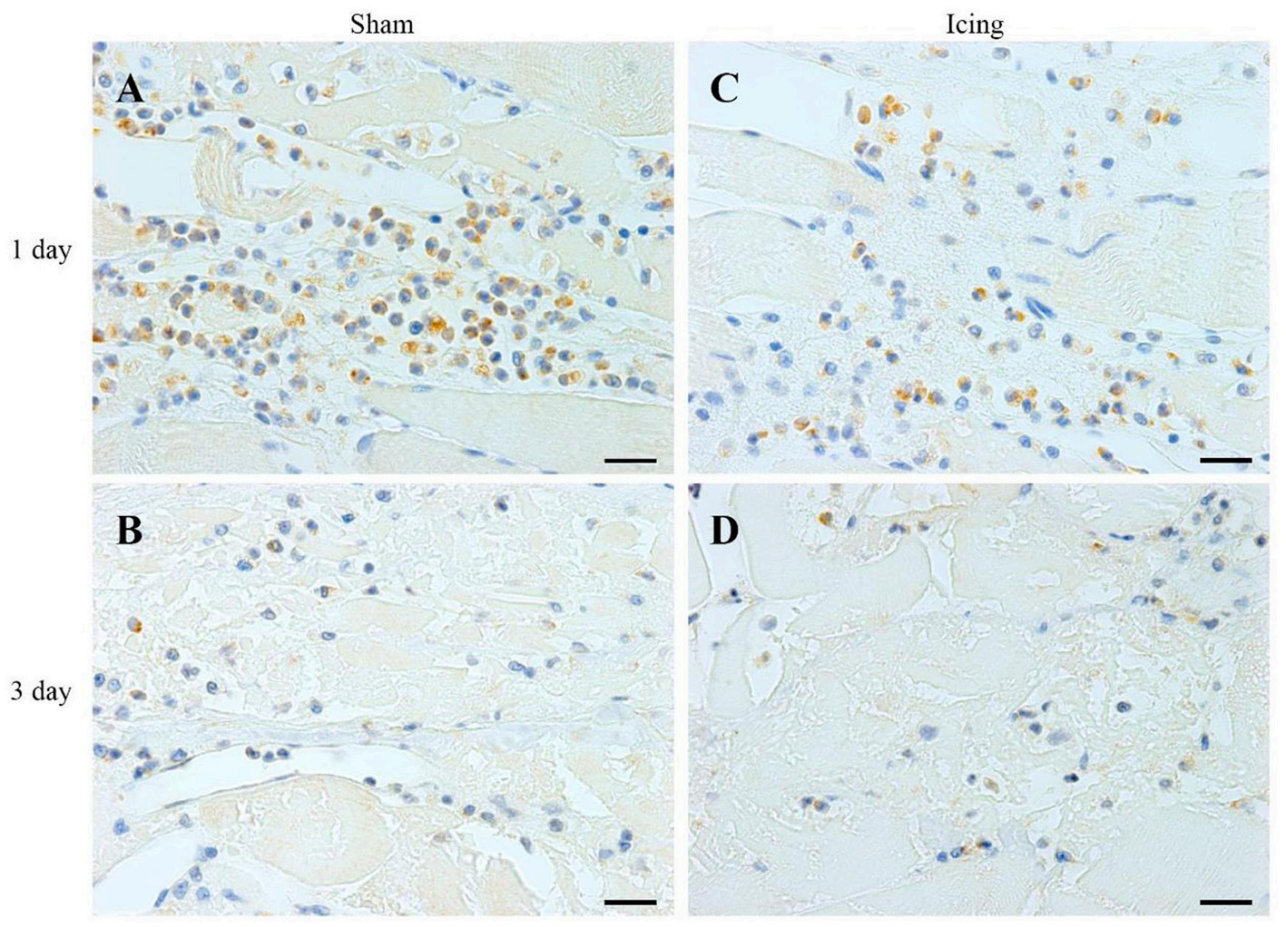
**Representative images of immunohistochemistry staining of neutrophils**. Skeletal muscle tissue was stained with HIS48 antibody to identify neutrophils. Sham **(A,C)** and icing **(B,D)** groups at 1 and 3 d after injury. Scale bar = 20 μm.

**Figure 6:**
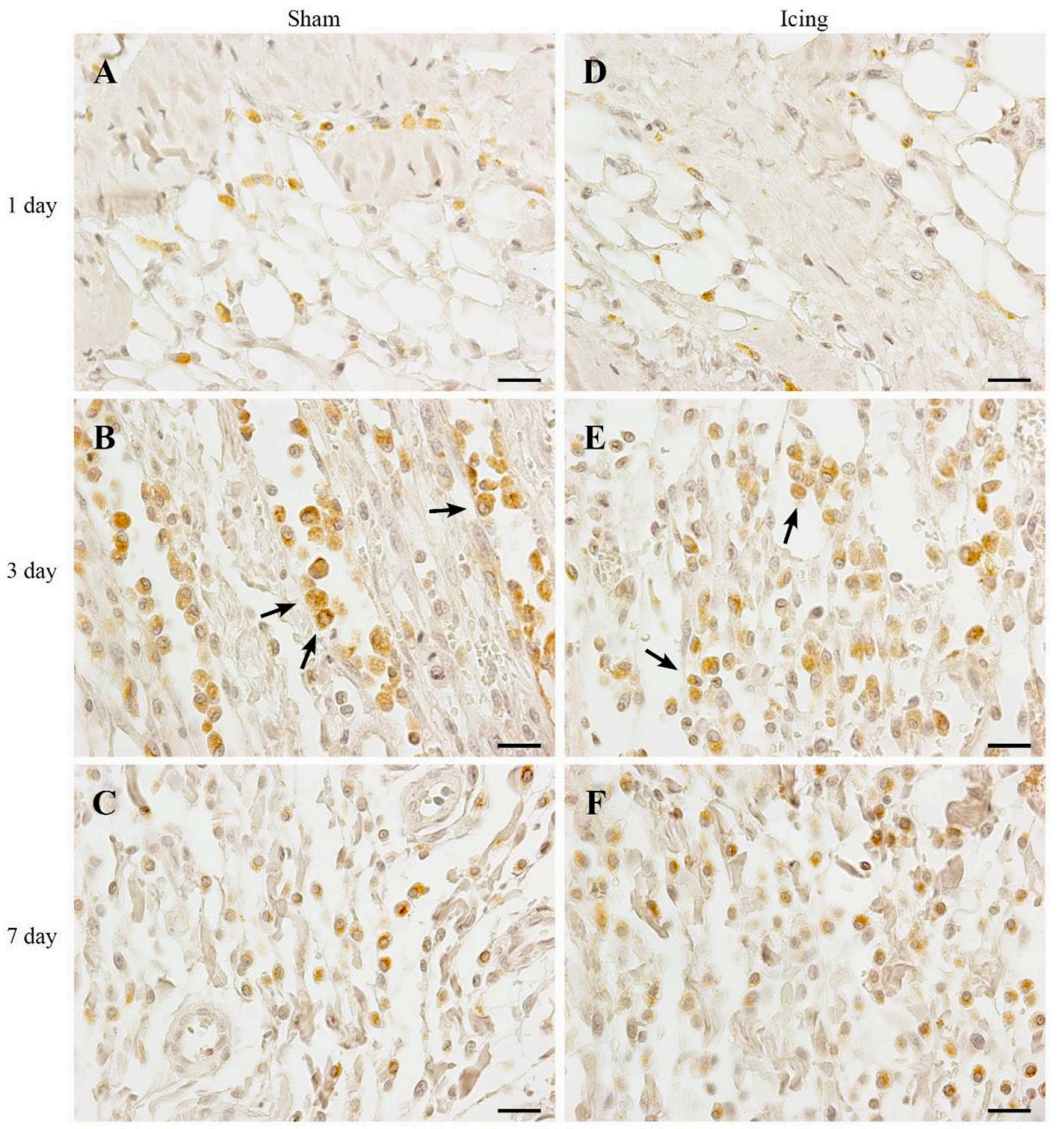
**Representative images of immunohistochemistry staining of macrophages**. Skeletal muscle tissue was stained with CD68 antibody to identify macrophages. Sham **(A–C)** and icing **(D–F)** groups at 1, 3, and 7 d after injury. Arrows indicate macrophages. Scale bar = 20 μm.

Routine histology with H&E staining revealed extensive necrosis of muscle fibers 1 d after injury in both the sham and icing groups (Figure 4). The necrotic muscle fibers were identified by enlarged myofibers without nuclei. Inflammatory cell infiltration accompanied the necrosis and comprised mainly multinucleated leukocytes (neutrophils) (Figure 5). In the rats sacrificed at 3 d after injury, the necrosis had cleared almost entirely in the sham group, whereas several necrotic areas were still present within the icing group. The inflammatory infiltrate at this time consisted mainly of mononuclear leukocytes, which were identified as macrophages by their size and single nuclei (Figure 6). Several macrophages were visible throughout the necrotic tissue in the sham group, but these cells were not as prominent in the icing group. In the rats sacrificed at 7 d after injury, the necrosis had cleared, and several immature centrally nucleated muscle fibers were evident in muscle tissue from both the sham and icing groups. In the rats sacrificed at 28 d after injury, the inflammatory cell influx had resolved, and normal tissue structure was almost restored with the exception of large maturing myofibers.

**Figure 7:**
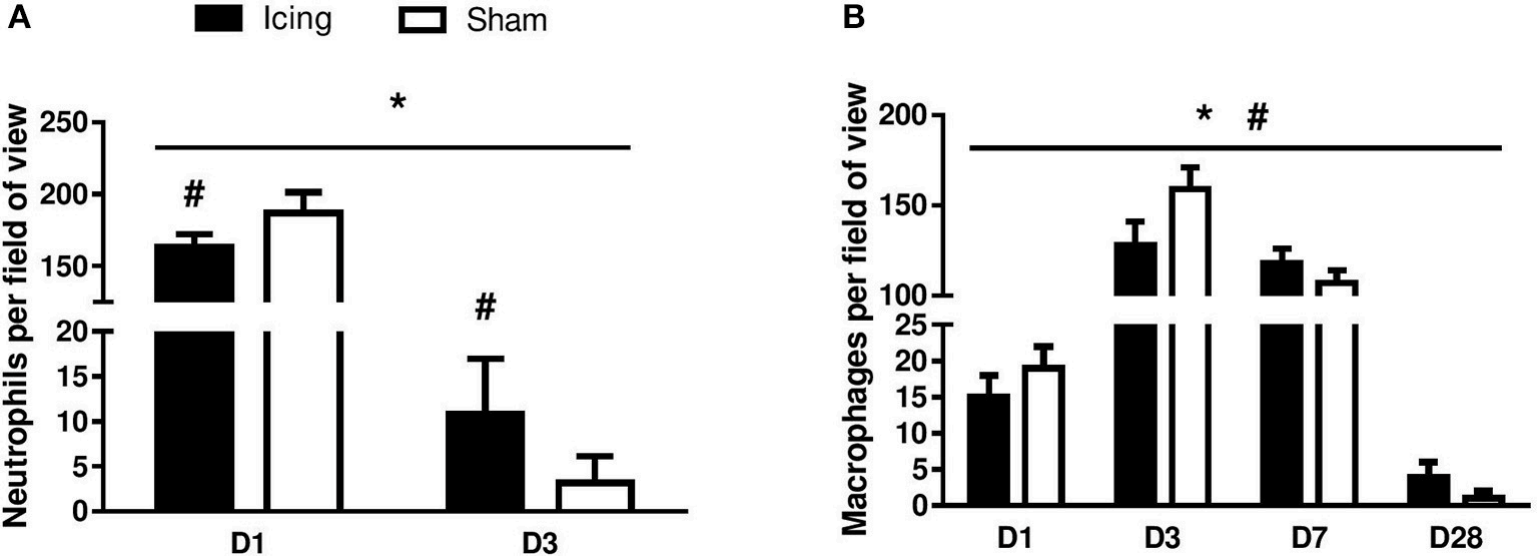
**Quantitative data for neutrophil (A)** and macrophage **(B)** count in skeletal muscle tissue. *n* = 6 rats per group. Data are mean ± SD and are expressed as a fold-difference from uninjured control rats. Neutrophils and macrophages were not present in skeletal muscle tissue from uninjured control rats. Main effects for neutrophil counts: time (*P* < 0.001), group (*P* = 0.029), and group × time interaction (*P* < 0.001). Main effects for macrophage counts: time (*P* < 0.001), group (*P* = 0.008), and group × time interaction (*P* < 0.001). ^*^P < 0.05 for unpaired *t*-test vs. uninjured control rats. #*P* < 0.05 for unpaired *t*-test vs. sham-treated rats.

The numbers of neutrophils were greater in muscle from the icing and sham groups compared with the control group at 1 d and 3 d after injury (Figure 7A). Neutrophils were more abundant in muscle from the sham group than in the icing group at 1 d after injury (*d* = 1.9; *P* = 0.010), whereas neutrophils were more abundant in muscle from the icing group than in the sham group at 3 d after injury (*d* = 1.6; *P* = 0.027). There were no neutrophils present in muscle in either group at 7 and 28 d after injury (data not shown).

The number of macrophages was greater in muscle from the icing and sham groups compared with the control group at all time points after injury (Figure 7B). Macrophages were more abundant in muscle from the sham group than in the icing group at 1 d (*d* = 2.0; *P* = 0.035) and 3 d after injury (*d* = 2.5; *P* = 0.001). By contrast, macrophages were more abundant in muscle from the icing group than in the sham group at 7 d (*d* = 1.5; *P* = 0.045) and 28 d (*d* = 1.9; *P* = 0.020) after injury.

**Figure 8:**
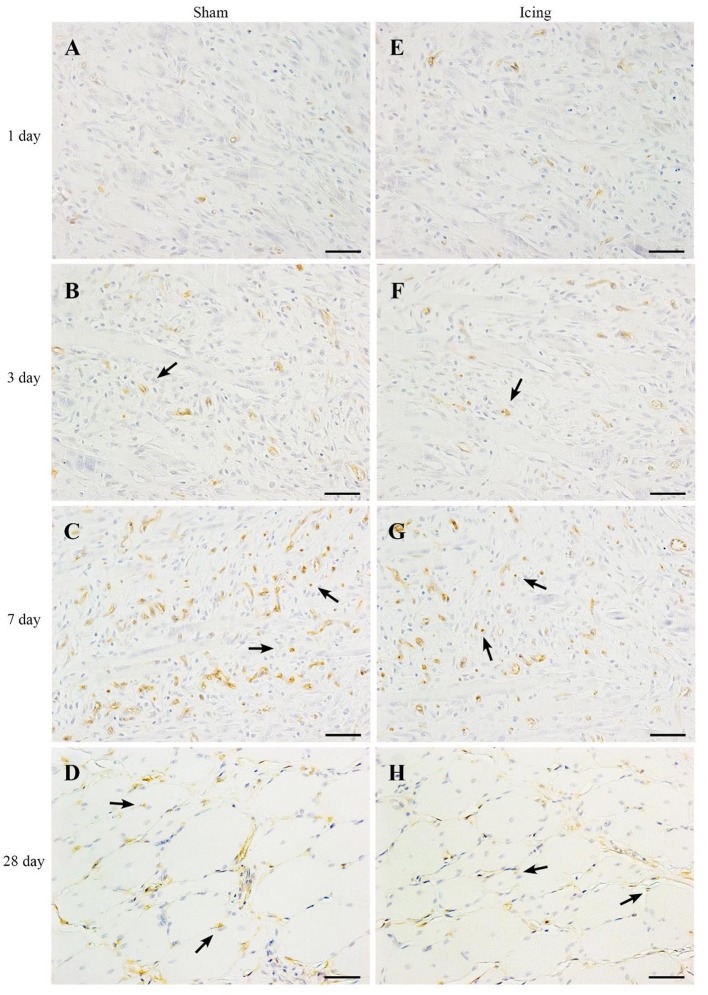
**Representative images of immunohistochemistry staining of endothelial cells. Skeletal muscle tissue was stained with CD34 antibody to identify endothelial cells**. Sham **(A–D)** and icing **(E–H)** groups at 1, 3, 7, and 28 d after injury. Arrows indicate vessels. Scale bar = 50 μm.

**Figure 9:**
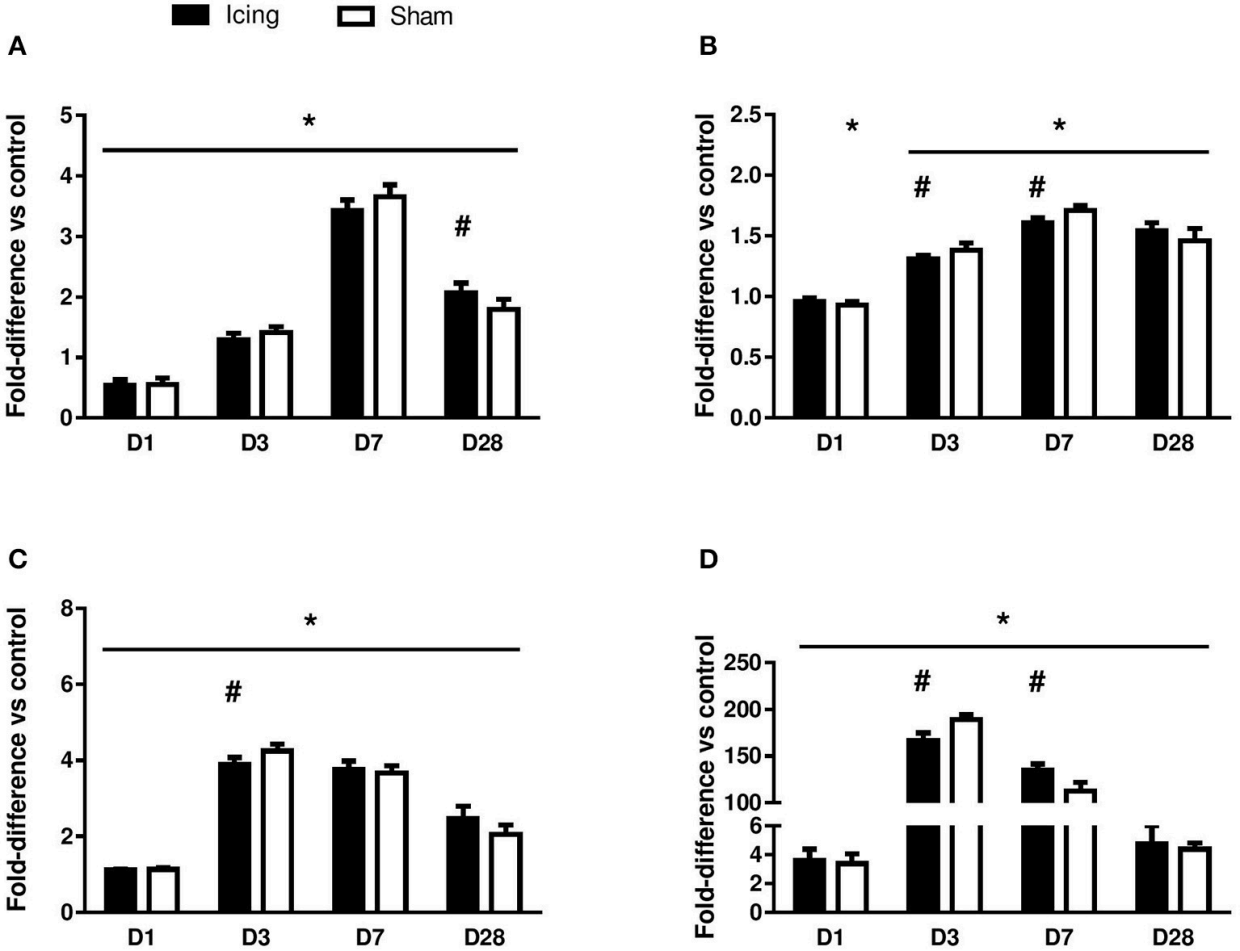
**Quantitative data for the area of positive staining for CD34 (A)**, vWF **(B)**, VEGF **(C)**, and nestin **(D)**. The data are shown as a percentage relative to the total area of muscle tissue within the field of view. Data are mean ± SD, *n* = 6 rats per group, and are expressed as a fold difference relative to the uninjured control rats. Main effects for CD34: time effect (*P* < 0.001), group effect (*P* < 0.001), and group × time interaction (*P* < 0.001). Main effects for vWF: time effect (*P* < 0.001), group effect (*P* = 0.63), and group × time interaction (*P* < 0.001). Main effects for VEGF: time effect (*P* < 0.001), group effect (*P* = 0.56), and group × time interaction (*P* < 0.001). Main effects for nestin: time effect (*P* < 0.001), group effect (*P* = 0.98), and group × time interaction (*P* < 0.001). ^*^P < 0.05 for unpaired *t*-test vs. uninjured control rats. #*P* < 0.05 for unpaired *t*-test vs. sham-treated rats.

CD34 staining was used to identify endothelial cells (Wiik et al., 2005; Ho et al., 2006; Hollemann et al., 2008) (Figure 8). Staining for CD34 was quantified as the area of positive staining as a percentage of the total area of tissue within the field of view. The percentage of CD34-stained area was lower in muscle from the icing and sham groups than in the control group at 1 d after injury (*P* < 0.001) (Figure 9A). Thereafter, the percentage of CD34-stained area was greater in muscle from the icing and sham groups than in the control group. The percentage of CD34-stained area tended to be greater in muscle from the sham group than in the icing group at 3 d (*d* = 1.1; *P* = 0.082) and 7 d (*d* = 1.3; *P* = 0.053) after injury. Conversely, the percentage of CD34-stained area was greater in muscle from the icing group than in the sham group at 28 d after injury (*d* = 1.7; *P* = 0.013).

**Figure 10:**
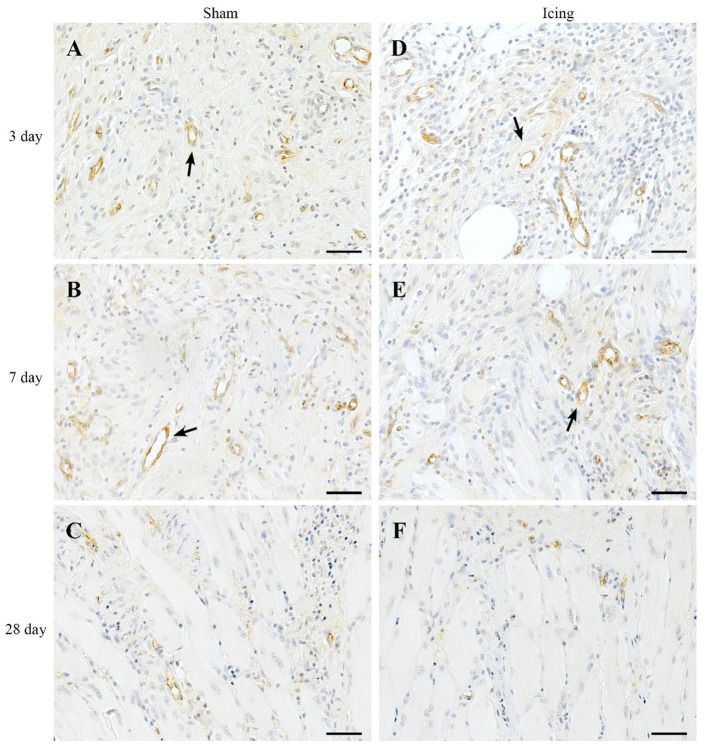
**Representative images of immunohistochemistry staining for vWF antibody to identify endothelial cells in muscle tissue**. Sham **(A–C)** and icing **(D–F)** groups at 1, 3, 7, and 28 d after injury. Arrows indicate mature vessels. Scale bar = 50 μm.

vWF staining was used to identify endothelial cells (Qu et al., 1997; Fujino et al., 2005; Hollemann et al., 2008). In contrast to the staining for CD34 and consistent with other reports (Qu et al., 1997), vWF staining was restricted to larger, well-established mature vessels (Figure 10). Staining for vWF was quantified as the area of positive staining as a percentage of the total area of tissue within the field of view. The percentage of vWF-stained area was greater in muscle from the icing and sham groups than in the control group at all time points after injury (Figure 9B), and this percentage was greater in muscle from the sham group than in the icing group at 3 d (*d* = 1.6; *P* = 0.022) and 7 d (*d* = 3.0; *P* < 0.001) after injury.

**Figure 11:**
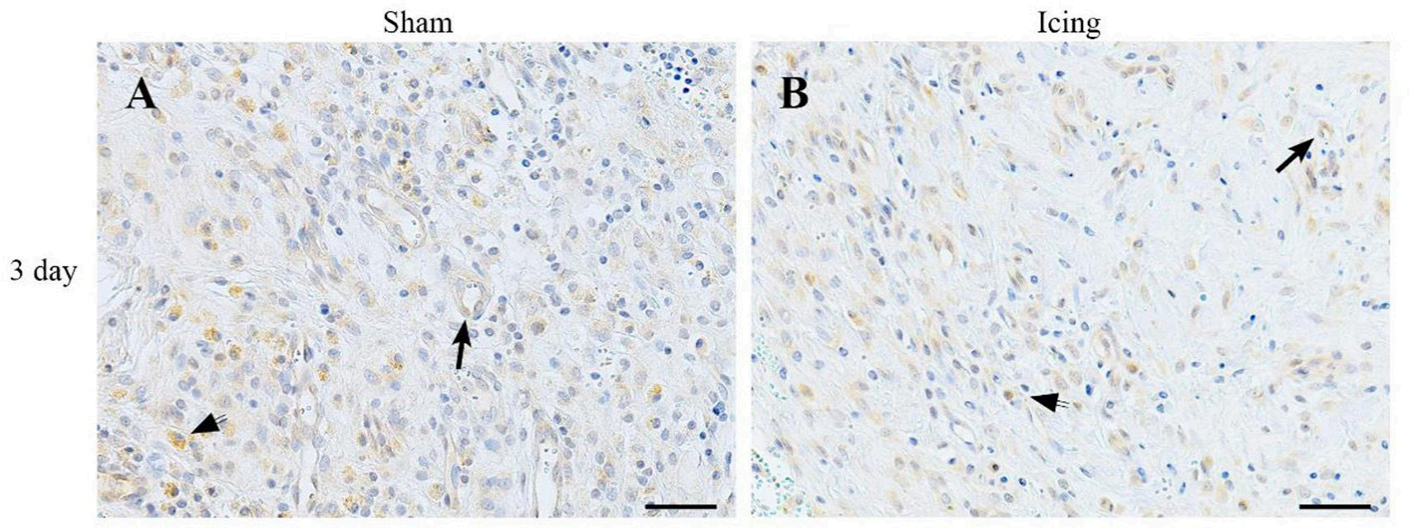
**Representative images of immunohistochemistry staining with VEGF antibody. Muscle tissue was obtained from the sham (A)** and icing **(B)** groups at 3 d after injury. Arrows indicate vessels expressing VEGF. Arrowheads indicate positively stained macrophages. Scale bar = 50 μm.

Broad background staining for VEGF was apparent in muscle tissue, and foci of VEGF staining were evident around blood vessels (Figure 11). The percentage of VEGF-stained area was greater in muscle from the icing and sham groups than in the control group at all time points after injury but appeared to peak at 3 d after injury (Figure 9C). It was greater in muscle from the sham group vs. the icing group at 3 d after injury (*d* = 2.0; *P* = 0.007).

**Figure 12:**
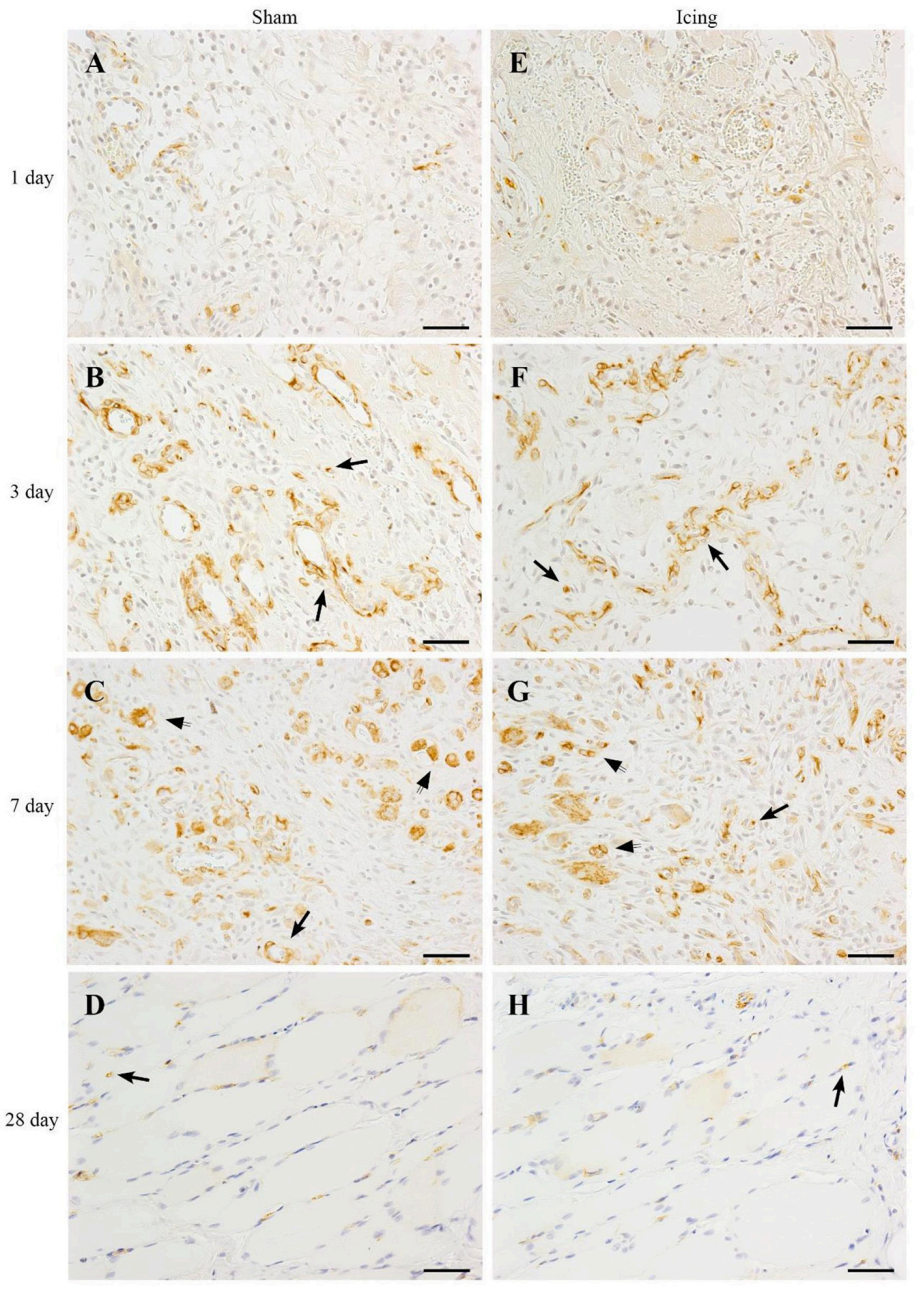
**Representative images of immunohistochemistry staining**. Nestin antibody was used to identify endothelial cells in muscle tissue from the sham **(A–D)** and icing **(E–H)** groups at 1, 3, 7, and 28 d after injury. Arrows indicate positively stained endothelial cells. Scale bar = 50 μm.

We used nestin to identify maturing endothelial cells (Cizkova et al., 2009b). Similar to vWF staining, nestin staining was localized to larger blood vessels (Figure 12). Staining for nestin was quantified as the area of positive stain as a percentage of the total area of tissue within the field of view. The area of nestin staining was greater in muscle from the icing and sham groups than in the control group at all time points after injury (Figure 9D). The area of nestin staining was greater in muscle from the sham group than in the icing group at 3 d after injury (*d* = 3.2; *P* < 0.001) but was greater in muscle from the icing group than in the sham group at 7 d (*d* = 2.7; *P* = 0.001) after injury. Nestin was also detected in the sarcoplasm of immature myotubes and myofibers. At 28 d, few large muscle fibers expressed nestin.

**Figure 13:**
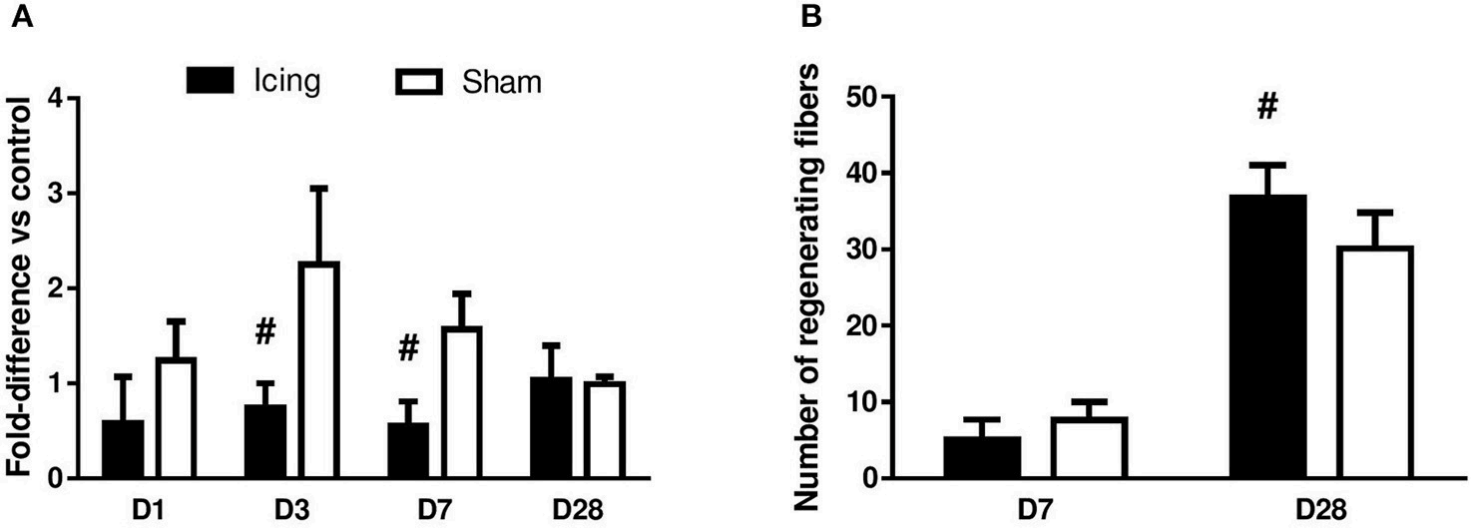
**Quantitative data for vessel volume (A)** and number of regenerating myofibers **(B)** in skeletal muscle tissue. Data are mean ± SD. Data for vessel volume were obtained from *n* = 4 rats per group and are expressed as a fold difference relative to the uninjured control rats. Data for number of regenerating myofibers were obtained from *n* = 6 rats per group. Main effects for vessel volume: time effect (*P* = 0.098), group effect (*P* < 0.001), and group × time interaction (*P* < 0.001). ^#^*P* < 0.05 for unpaired *t*-test vs. sham-treated rats.

The vessel volume in muscle was determined by micro-CT. The vessel volume was greater in the sham group than in the icing group at 3 d (*d* = 2.5; *P* = 0.029) and 7 d (*d* = 3.2; *P* = 0.021) after injury, whereas there was no difference between the groups after 28 d (*P* = 0.84) (Figure 13A). The number of capillaries per fiber at 28 d after injury did not differ significantly (*P* = 0.59) between the icing group (1.90 ± 0.27) and the sham group (1.82 ± 0.18). The number of capillaries per millimeter squared also did not differ significantly (*P* = 0.13) between the icing group (240 ± 24) and the sham group (218 ± 0.23) at 28 d after injury.

At 7 d after injury, many centrally nucleated regenerating muscle fibers were present in the sham group, whereas only a few were present within the icing group. The percentage of regenerating fibers relative to the total number of fibers did not differ significantly between the icing and sham groups at 7 d after injury (*P* = 0.11). By contrast, this percentage was greater in the icing group than in the sham group at 28 d after injury (*d* = 1.5; *P* = 0.026) (Figure 13B). There were no significant differences between the icing and sham groups for fiber cross-sectional area at 7 d (*P* = 0.35) or 28 d (*P* = 0.30) (data not shown).

## Discussion

The aim of this study was to examine the effects of icing soon after muscle contusion injury on subsequent inflammation, angiogenesis, revascularization, and myofiber regeneration. Our study is the first to demonstrate that icing delayed and/or attenuated the expression of proangiogenic factors and changes in vessel volume in regenerating muscle in the first 7 d after injury. Despite these differences, capillary density and the cross-sectional area of myofibers did not differ significantly between the icing and sham groups. These findings suggest that, although icing may mildly suppress inflammation and some aspects of angiogenesis/revascularization, these effects are not sufficient to retard muscle regeneration after contusion injury.

Contusion injury caused extensive necrosis in skeletal muscle during the first 3 d after injury. Icing appeared to prolong the clearance of necrotic tissue, possibly by preventing (or delaying) the infiltration of neutrophils and macrophages into the damaged muscle (Teixeira et al., 2003; Summan et al., 2006). The decline or delay in infiltration of inflammatory cells is consistent with the results of other studies that have treated muscle injuries with ice (Carvalho et al., 2010; Puntel et al., 2011; Takagi et al., 2011) or cold saline (Lee et al., 2005; Schaser et al., 2006, 2007). This decline/delay in inflammation may result from a decrease in the expression of adhesion molecules on the surface of inflammatory cells (Haddix et al., 1996; Inamasu et al., 2001).

This is the first study to investigate whether icing influences angiogenesis and vessel volume in regenerating muscle. We stained muscle tissue for CD34 and vWF to identify capillaries (Qu et al., 1997; Niiyama et al., 2003; Fujino et al., 2005; Wiik et al., 2005; Ho et al., 2006; Hollemann et al., 2008). Contusion injury induced a sustained increase in the expression of vWF in muscle that lasted at least 28 d. Conversely, contusion injury appeared to suppress CD34 expression in muscle in the first 7 d after injury. Icing reduced the expression of vWF and CD34 (to a lesser extent) between 3 and 7 d after injury. As reported by Ochoa et al. (2007), necrosis in the muscle tissue at 3 and 7 d after injury made it difficult to identify the border of muscle fibers and therefore to quantify the number of capillaries per fiber (i.e., capillary density). Nevertheless, we were able to quantify the area of positive staining for CD34, vWF, and VEGF as percentages of the total area of tissue within the field of view. Despite a difference in CD34 staining intensity in muscle after 28 d (Figure 9A), capillary density did not differ significantly between the icing and sham groups at this time point. However, it is possible that capillary density differed between these groups at 3 and 7 d after injury. Some VEGF staining was evident in endothelial cells and surrounding blood vessels (Figure 11), but there was also general background staining for VEGF, which most likely represents staining of muscle cells. As such, we cannot attribute the VEGF staining exclusively to angiogenesis.

These differences in staining for vWF and VEGF between the icing and sham groups were accompanied by smaller vessel volume at 3 and 7 d after injury, as measured by contrast-enhanced micro-CT (Figure 13A). Because we could not assess capillary density at these time points, it is difficult to compare the time courses of angiogenesis and collateral vessel growth, as assessed by vessel volume. Others have reported differences in the time course of angiogenesis and collateral vessel growth in skeletal muscle following hind limb ischemia (Ito et al., 1997; Hershey et al., 2001, 2003). It is possible that icing exerted a greater effect on vessel volume than on angiogenesis. The present findings contrast with previous research showing that perfusion of skeletal muscle with cold saline at 8°C restores functional capillary density and venule diameter 1 d after injury (Schaser et al., 2006, 2007). Our results are not directly comparable with this other research because we assessed static measures of angiogenesis, whereas Schaser et al. measured changes in microcirculatory dynamics in muscle using intravital microscopy (Schaser et al., 2006, 2007). Their assessment was also restricted to 1 d after injury. Icing may induce different effects on microcirculatory dynamics in muscle in the days and weeks after injury.

Various factors or mechanisms may explain why icing attenuated and/or delayed the expression of VEGF and vWF in regenerating muscle tissue. Icing delays satellite cell proliferation in damaged muscle (Takagi et al., 2011), which may also partly explain why icing reduced the expression of VEGF and vWF in the current study. Evidence for this notion is that proliferating and differentiating satellite cells stimulate human vascular endothelial cells to form tubular-like structures *in vitro* (Christov et al., 2007), possibly by secreting VEGF and expressing HIF-1α (Rhoads et al., 2009). Skeletal muscle expresses cold shock domain protein A (Saito et al., 2011). This protein suppresses VEGF promoter activity and VEGF secretion by skeletal muscle cells under normoxic and hypoxic conditions (Saito et al., 2011) and suppresses tube formation by endothelial cells (Saito et al., 2011). Although it is unknown whether icing influences the expression of cold shock domain protein A in skeletal muscle, it is possible that this protein may have been partially responsible for reducing/delaying VEGF and vWF expression in the present study.

Incubation of human vascular endothelial cells under “hypothermic” conditions (i.e., ≤34°C) reduces their capacity to form tubes *in vitro* (Coassin et al., 2010; Takeyama et al., 2015). Hypothermia also significantly reduces VEGF secretion and mRNA expression by retinal pigment epithelial cells (Coassin et al., 2010; Takeyama et al., 2015). These effects appear to be linked to lower cellular metabolism (Coassin et al., 2010; Takeyama et al., 2015). When amputated, ischemic limbs of cats are exposed to hypothermia, ATP and phosphocreatine depletion and tissue acidosis are attenuated (Osterman et al., 1984; Sapega et al., 1988).

The effects of hypothermia on the production of reactive oxygen species (ROS) are variable (Rauen and de Groot, 2002; Alva et al., 2013b). Some research demonstrates that when applied in isolation, hypothermia increases the production of ROS by adenocarcinomic alveolar basal epithelial (A549) cells (Sun et al., 2016) and induces lipid peroxidation and decreases the blood concentration of glutathione in rats (Dede et al., 2002). Conversely, many studies have reported that when applied following injury, hypothermia reduces the formation of ROS in neuronal cells (Gao et al., 2014, 2016), PC12 adrenal cells (Hasegawa et al., 2009), brain tissue (Maier et al., 2002; Horiguchi et al., 2003; Lv et al., 2016) liver tissue and blood (Alva et al., 2013a). In injured rat skeletal muscle, icing decreases the formation of superoxide anions, lipid peroxidation, and activity of the antioxidant enzyme catalase (Merrick et al., 1999; Carvalho et al., 2010). These findings contrast with other evidence that hypoxia stimulates ROS production in skeletal muscle fibers (Zuo and Clanton, 2005; Zuo et al., 2013b). The effects of hypothermia or cryotherapy on ROS production by cells and tissues may therefore depend on various factors including the temperature at which cells and tissues are treated, the extent of ischemia and hypoxia that occurs in response to hypothermia or cryotherapy, and whether these treatments are applied to cells/tissues in isolation or after injury (Alva et al., 2013b; Zuo et al., 2013a). We did not measure ROS formation in muscle in the present study, but it is tempting to speculate that icing may have reduced the expression of VEGF and vWF and vessel volume in skeletal muscle by reducing ROS production (Merrick et al., 1999; Carvalho et al., 2010). However, other evidence indicates that hypothermia suppresses the expression of VEGF (and HIF-1α) by T98G cells (derived from human glioblastoma multiform) independently of changes in O_2_ consumption (Tanaka et al., 2010). Evidence also exists that hypothermia increases angiogenesis in the spinal cord (Kao et al., 2011) and brain (Kuo et al., 2010). More research is therefore needed to understand the effects of icing on ROS production and angiogenesis in skeletal muscle following injury.

We measured the expression of nestin as a marker of revascularization in skeletal muscle (Cizkova et al., 2009b). Nestin has been detected in endothelial cells (Mokry et al., 2004; Cizkova et al., 2009b), pericytes (Birbrair et al., 2014), and blood vessels (Amoh et al., 2005) within skeletal muscle. Consistent with other research (Vaittinen et al., 2001; Cizkova et al., 2009a,b), we observed that nestin expression was greatest in regenerating muscle tissue between 3 and 7 d after injury. At 3 d after injury, there appeared to be strong nestin staining around blood vessels (Figure 12), suggesting that nestin was expressed by maturing endothelial cells (Cizkova et al., 2009b). Pericytes also express nestin, colocalize with endothelial cells and capillaries in skeletal muscle, and contribute to angiogenesis (Birbrair et al., 2014). We found that icing attenuated or delayed upregulation of nestin expression in regenerating muscle. Considering that nestin is involved in the differentiation of endothelial cells (Cizkova et al., 2009b), suppression of nestin expression following icing may also have contributed to the reduction in vWF expression and vessel volume that we observed. We did not double-stain the muscle tissue for nestin and another marker of endothelial cells (e.g., CD34, vWF) or pericytes (e.g., NG2). Nor did we stain the muscle for myotubes, Schwann cells, or axons, which also express nestin (Cizkova et al., 2009b). Accordingly, we cannot establish definitively whether the nestin staining that we observed solely represents revascularization.

We found that icing did not alter myofiber cross-sectional area at 7 or 28 d after injury. However, it did appear to delay myofiber maturation, as indicated by a greater number of immature myofibers with centrally located nuclei at 28 d after injury (Figure 13B). Our findings contrast with those of Takagi et al, who reported that icing resulted in more immature myofibers at 14 d (but not at 28 d) and smaller myofiber cross-sectional area at 28 d after injury (Takagi et al., 2011). These differences may be explained by the use of younger rats (8 weeks old) and induction of injury by crushing the *extensor digitorum longus* muscle with forceps for 30 s in the study by Takagi et al. Collectively, these factors may have resulted in a different degree of muscle injury and/or rate of muscle repair between studies, which may in turn have influenced the efficacy of the icing treatment.

In conclusion, icing attenuated or delayed the infiltration of inflammatory cells, the expression of proangiogenic factors, and change in vessel volume in muscle following injury. However, these effects were not sufficient to reduce capillary density or prevent effective muscle regeneration. We applied ice treatment at only one time point soon after muscle injury. It is possible that more frequent icing may have produced different effects on inflammation, angiogenesis, and myofiber regeneration. We also used only male rats. Icing may have produced different effects in female rats because of the effects of estrogen on the time course and dynamics of muscle regeneration (Enns and Tiidus, 2010). Future research could systematically examine whether the duration, timing, or frequency of icing treatment influence inflammation, angiogenesis, and muscle tissue regeneration after injury. Also of interest is whether icing influences the expression/activity of antiangiogenic factors such as endostatin and angiostatin or the release of neurogenic inflammatory factors (e.g., bradykinin, nerve growth factor, calcitonin gene-related peptide) that regulate pain responses in muscle following injury.

## Author contributions

DS, ZB, MW, TP, RS, and JP designed the study. DS, ZB, and RS performed the experiments. DS, ZB, RS, and JP analyzed and interpreted the data. DS, ZB, MW, TP, RS, and JP drafted and revised the work. DS, ZB, MW, TP, RS, and JP approved the final version. DS, ZB, MW, TP, RS, and JP agree to be accountable for all aspects of the work in ensuring that questions related to the accuracy or integrity of any part of the work are appropriately investigated and resolved.

## Funding

This work was funded by a grant from the Institute of Health and Biomedical Innovation at Queensland University of Technology.

### Conflict of interest statement

The authors declare that the research was conducted in the absence of any commercial or financial relationships that could be construed as a potential conflict of interest.

## Abbreviations

CT: computed tomography
HIF-1α: hypoxia-inducible factor 1α
ROS: reactive oxygen species
vWF: von Willebrands factor
VEGF: vascular endothelial growth factor.
